# Cost analysis of an active surveillance strategy for *Clostridium difficile* using an agent-based simulation

**DOI:** 10.1186/2047-2994-4-S1-P21

**Published:** 2015-06-16

**Authors:** M Rubin, M Leecaster, W Ray, R Nelson, K Khader, D Toth, V Stevens

**Affiliations:** 1 University of Utah School of Medicine, Salt Lake City, USA; 2 Department of Veterans Affairs, Salt Lake City, USA

## Introduction

*Clostridium difficile* (CD) is one of the most common nosocomial infections in the United States. Rapid identification and isolation of hospitalized patients with CD colonization or infection is critical to preventing transmission.

## Objectives

This work assesses the potential impact of an active surveillance (AS) strategy for CD via admission testing on overall costs and infections.

## Methods

We designed an agent-based simulation of nosocomial CD transmission with static and dynamic components including: patients, healthcare workers (HCW), and rooms; patient admission, discharge, and transfer; interactions between HCW and patients; contamination of rooms by patients shedding CD; HCW hand carriage and removal via hand hygiene or prevention via personal protective equipment; and patient acquisition of CD following contact with contaminated rooms or HCW. Model parameters were derived from local data, literature where available, and expert opinion. The model was calibrated against local data and validated internally and externally.

Two scenarios with varying CD importation prevalence were simulated 60 times each over a one-year period. One scenario reflected the usual strategy of no admission testing, while another reflected an AS strategy with CD testing of all patients upon admission. Importation prevalence was varied in a sensitivity analysis from 0%>30%. Cost input parameters were obtained from the literature.

## Results

At moderate to high levels of CD importation (≥9%) an AS strategy is cost-saving overall, reducing costs by between $10 and $20 per patient, owing primarily to CD infections prevented. The cost savings plateaued at an importation prevalence above 12%. At low CD importation (<6%), the AS strategy costs more than no surveillance, though costs per patient are low. Up to 38 CD infections per 10,000 patient-days could be prevented with an AS strategy.

## Conclusion

Despite the additional cost of testing and isolation, an inpatient AS strategy for CD may be cost-saving through the prevention of CD transmissions and infections. Given reported CD admission prevalence and infection mortality rates, further consideration of this strategy may be worthwhile.

## Disclosure of interest

None declared.

